# Factors associated with frailty in older adults: a longitudinal study

**DOI:** 10.11606/S1518-8787.2018052000497

**Published:** 2018-07-13

**Authors:** Jack Roberto Silva Fhon, Rosalina Aparecida Partezani Rodrigues, Jair Lício Ferreira Santos, Marina Aleixo Diniz, Emanuella Barros dos Santos, Vanessa Costa Almeida, Suelen Borelli Lima Giacomini

**Affiliations:** I Universidade de São Paulo. Escola de Enfermagem de Ribeirão Preto. Departamento de Enfermagem Geral e Especializada. Ribeirão Preto, SP, Brasil; II Universidade de São Paulo. Faculdade de Medicina de Ribeirão Preto. Departamento de Medicina Social. Ribeirão Preto, SP, Brasil

**Keywords:** Aged, Frail Elderly, Risk Factors, Socioeconomic Factors, Aging, Idoso, Idoso Fragilizado, Fatores de Risco, Fatores Socioeconômicos, Envelhecimento

## Abstract

**OBJECTIVE:**

To determine the demographic and health factors related to the frailty syndrome in older adults.

**METHODS:**

This is a longitudinal quantitative study carried out with 262 older adults aged 65 years and older, of both sexes, living at home. Data collection was carried out in Period 1 between October 2007 and February 2008, and in Period 2 between July and December 2013. For data collection, we used the sociodemographic profile instrument, the Edmonton Frail Scale, the Mini-Mental State Examination, the number of falls in the last 12 months, the number of self-reported diseases and used drugs, the Functional Independence Measure, and the Lawton and Brody Scale. We used descriptive statistics for data analysis, in the comparison of the means between periods, the nonparametric Wilcoxon test, and the method of Generalized Estimating Equations, which is considered an extension of the Generalized Linear Models with p ≤ 0.05.

**RESULTS:**

Of the 515 participants, 262 completed the follow-up, with a predominance of females, older individuals, and those who had no partner; there was an increase in frail older adults. In the Generalized Estimating Equations analysis, frailty score was related to sociodemographic (increase in age, no partner, and low education level) and health variables (more diseases, drugs, falls, and decrease in functional capacity). There was an association between the variables of age (older), marital status (no partner), and loss of functional capacity.

**CONCLUSIONS:**

Frailty syndrome was associated with increasing age, having no partner, and decreased functional capacity over time, and investments are required to prevent this syndrome and promote quality in aging.

## RESUMO

**OBJETIVO:**

Determinar os fatores demográficos e de saúde relacionados com a síndrome da fragilidade em idosos.

**MÉTODOS:**

Estudo quantitativo longitudinal, realizado com 262 idosos acima de 65 anos de ambos os sexos, que vivem no domicílio. A coleta das informações no Tempo 1 foi realizada entre outubro de 2007 e fevereiro de 2008, e no Tempo 2 entre julho e dezembro de 2013. Para a coleta das informações, foi utilizado o instrumento do perfil sociodemográfico, a *Edmonton Frail Scale*, o Mini Exame do Estado Mental, o número de quedas nos últimos 12 meses, o número de doenças autorreferidas e medicamentos, a Medida de Independência Funcional e a Escala de Lawton e Brody. A estatística descritiva foi utilizada para a análise de dados, na comparação das médias entre ambos os tempos, o Teste não paramétrico de Wilcoxon e o método de Equações de Estimação Generalizadas, considerado uma extensão dos Modelos Lineares Generalizados com p ≤ 0,05.

**RESULTADOS:**

Dos 515 participantes, 262 completaram o seguimento, com predomínio do sexo feminino, idosos mais velhos, sem companheiro(a); houve aumento de idosos frágeis. Na análise Equações de Estimação Generalizadas, o escore da fragilidade teve relação com as variáveis sociodemográficas (aumento da idade, estado civil sem companheiro(a) e baixa escolaridade), e de saúde (maior número de doenças, medicamentos, queda e diminuição da capacidade funcional). Verificou-se associação com as variáveis idade (mais velhos), o estado conjugal (não ter companheiro), e perda da capacidade funcional.

**CONCLUSÕES:**

A síndrome da fragilidade esteve associada ao aumento da idade, estar sem companheiro(a) e diminuição da capacidade funcional ao longo do tempo, sendo necessários investimentos para a prevenção dessa síndrome e promoção de um envelhecimento de qualidade.

## INTRODUCTION

The frailty syndrome is the decrease of the homeostatic reserves of the organism and resistance to stressors. It results in a decline of the physiological system^1^, based on the triad of the different changes related to the aging process, consisting of sarcopenia, immunological dysfunction, and neuroendocrine dysregulation^2^.

The multidimensional construct with a holistic approach is established or altered by biological, psychological, and social factors. It emphasizes the existing complex etiology, which is understood as a non-optimal condition of multifactorial and dynamic nature related to history or life trajectory^3^.

A group of six European and American societies has defined frailty as: “a medical syndrome with multiple causes and contributors that is characterized by diminished strength, endurance, and reduced physiologic function that increases an individual’s vulnerability for developing increased dependency and/or death”^4^ (p.4).

Frailty is related to demographic factors, such as being female, increasing age^5^, and the presence of adverse health events, such as decreased cognitive status^6^, polypharmacy^7^, sarcopenia^8^, falls^9^, institutionalization, hospitalization^10^, and death^11^.

The changes that occur in an individual during the aging process encompass physical, psychic, and social aspects that make him or her more vulnerable. The increase in life expectancy has become a victory for society because of improvements in health services and accessibility to them. Nurses need constant training in the care for the older population to respond to the demands of frail older adults, which can go unnoticed and shorten their life expectancy. The evaluation of the older adult in primary care is also up to the nurse; thus, new possibilities to evaluate this population are available in the literature, which enables the planning of care for the older adult at home. The objective of this study was to determine the demographic and health factors related to the frailty syndrome in older adults.

## METHODS

This is a longitudinal study carried out in the city of Ribeirão Preto, State of São Paulo, Brazil. Data collection was carried out in Period 1 (P1), the first collection, between October 2007 and February 2008, and in Period 2 (P2), the second collection, between July and December 2013.

We used the probabilistic method and conglomerate in two stages for the sampling process. In the second stage, we made home visits to ensure a self-weighting sample. We randomly selected the street and block of the tracts, and we needed to visit at least 110 households in each tract. We randomly selected 993 older adults; but the final sample was 515 (51%) participants.

Inclusion criteria in P1 were: being an older adult aged ≥ 65 years, both sexes, being able to communicate verbally, and living at home. For P2, they were: having participated in P1 and being able to communicate.

Data collection in P1 followed the counterclockwise direction of the randomly selected blocks, and we eliminated from the sample the older adult who was not at home after three visits. For P2, we contacted the older adult or family member by telephone to arrange a home visit or the visit to the address in order to schedule the interview according to the availability of the older adult.

The sample consisted of 515 older adults in P1 and 262 older adults in P2. Among the losses, in the five years, 24.7% of them died, 16.1% refused to participate in the research, 1.6% was institutionalized, and 6.8% moved to other cities.

We used the following instruments:

- Demographic profile, with information on sex (male and female), age (in years), marital status, and education level (in years of formal study);
- Self-reported diseases and number of drugs, from the self-report of the participant and the checking of drugs and medical prescription;
- Edmonton Frail Scale (EFS), used to evaluate the frailty syndrome^12^ and validated and reproduced in Brazilian Portuguese^13^ ^,^ ^14^ with nine domains represented in eleven items. According to the cut-off point, the older adult can be categorized as: not frail (0–4), apparently vulnerable (5–6), mild frailty (7–8), moderate frailty (9–10), and severe frailty (11 or more);
- Instrument for falls, number of falls in the last 12 months;
- Mini-Mental State Examination (MMSE), used to evaluate the cognitive function, translated, validated, and revised for Brazilian Portuguese^15^, with questions grouped into seven categories and evaluation of different cognitive functions. Score ranges from zero to 30 points;
- Functional Independence Measure (FIM), developed in order to measure the degree of care need by the disabled patient to perform motor and cognitive tasks and reproduced^16^ and validated^17^ for Portuguese. It has a score ranging from one (complete dependence) to seven (complete independence); the total score varies from 18 to 126 points and the higher scores refer to greater independence;
- Lawton and Brody scale, validated for Portuguese^18^, it encompasses complex social activities. It evaluates the ability of the older adult to live in the community and measures the Instrumental Activities of Daily Living (IADL). The score varies from seven (highest level of dependency) to twenty-one points (complete independence), categorizing the older adult in total dependence (7 points), partial dependence (8–20 points), and independence (21 points).

We used descriptive statistics for quantitative data, as well as the measure of central tendency (mean and median) and dispersion (standard deviation); we used frequency and percentages for the categorical variables. We used the non-parametric Wilcoxon test to compare the means between P1 and P2.

In order to analyze the relationship between frailty and the sociodemographic (sex, age, education level, and marital status) and health variables (cognitive status, functional capacity, falls, and number of morbidities and drugs), we used the Generalized Estimating Equations (GEE). This method is considered an extension of the Generalized Linear Models (GLM), and it takes advantage of the assumptions of the existence of a mean link function with covariates and the response variable not needing to belong to the exponential family of distributions; it adds a correlation structure among repeated measures.

The dependent variable of the analysis was the frailty score, with integer values from zero to seventeen, following a Poisson distribution. The independent variables were age (in years), sex (male and female), marital status (with partner and without partner), education level (in years), falls (yes and no), total number of diseases, total number of drugs, cognitive status (with or without deficit), and scores of the FIM and IADL scales.

We calculated relative increase for the resulting final model based on the mean regression parameters: RI (β) = (EXP(β) – 1) × 100%, where β is the parameter vector of the adjusted model. For all statistical tests, significance was p ≤ 0.05.

The study was approved by the Research Ethics Committee of the Escola de Enfermagem de Ribeirão Preto of Universidade de São Paulo (Protocols 0851/2007 and 1392/2011) and the participants signed the informed consent.

## RESULTS

In the evaluation of frailty of the 262 older adults of the cohort, in P1, 17.6% of the participants were considered frail, 22.9% vulnerable, and 59.5% not frail. In P2, 50.4% were frail, 21.0% vulnerable, and 28.6% not frail. Those aged 80 years or more, females, those without a partner, and those with a lower mean education level were predominant compared to the other categories (Table 1). Mean frailty increased from 4.2 (standard deviation [SD] = 2.6) in P1 to 6.5 (SD = 3.1) in P2 with p < 0.001.

**Table 1:** Sociodemographic profile at the end of the follow-up of the older adults living in the community. Ribeirão Preto, State of São Paulo, Brazil, 2016.

Variable	Cut-off total	Frail	Vulnerable	Not frail
			
	n = 262	n = 132	n = 55	n = 75
Mean age (SD) (in years)	79.3 (6.3)	80.5 (6.8)	79.2 (6.2)	77.3 (5.1)
70 to 79	149 (56.9)	63 (47.7)	34 (61.8)	52 (69.3)
80 or more	113 (43.1)	69 (52.3)	21 (38.2)	23 (30.7)
Sex				
Female	174 (66.4)	91 (68.9)	37 (67.3)	46 (61.3)
Male	88 (33.6)	41 (31.1)	18 (32.7)	29 (38.7)
Marital status				
With a partner	106 (40.5)	45 (34.1)	18 (32.7)	43 (57.3)
Without partner	156 (59.5)	87 (65.9)	37 (67.3)	32 (42.7)
Mean education level (SD)	5.0 (4.9)	4.1 (4.8)	4.8 (4.5)	6.4 (5.1)

In the GEE analysis, the total frailty score was related to some sociodemographic variables such as age: for each additional year, we estimated a mean increase of 0.8% in the frailty score. The older adults who did not have a partner had a mean frailty score of 10.4%, higher than those with a partner. On the other hand, we estimated a decrease of 1.2% in the frailty score for each additional year of education. There was an increase in mean frailty score of 1.7% for each drug used by the older adult. Mean frailty score increased 14.1% for each fall that the older adult suffered and 3.2% for each disease present (Table 2).

**Table 2:** Adjustment obtained by analysis of generalized estimation equation for the frailty score. Ribeirão Preto, State of São Paulo, Brazil, 2016.

Variable	Estimate	Standard error	Wald	p*
Intercept	0.761	0.194	15.359	< 0.001
Age	0.007	0.194	15.359	< 0.001
Without partner	0.099	0.036	7.555	< 0.001
Education level	-0.011	0.004	7.608	0.005
Number of diseases	0.031	0.004	41.795	< 0.001
Number of drugs	0.016	0.005	8.387	0.003
Suffered a fall	0.135	0.034	14.327	< 0.001
Linear total FIM	-3.096	0.522	35.169	< 0.001
Quadratic total FIM	-0.915	0.347	6.945	0.008
Linear total IADL	-3.773	0.573	43.262	< 0.001
Quadratic total IADL	-2.219	0.357	38.620	< 0.001
Period 2 (2013)	-0.103	0.035	8.663	0.003
Dispersion (Intercept)	0.759	0.047	260.606	< 0.001
Correlation	0.207	0.060	11.628	< 0.001

FIM: functional independence measure; IADL: instrumental activities of daily living

* p ≤ 0.05

In the adjustment obtained by the GEE in the dispersion analysis, the relative increase in mean frailty was 0.5% at each year of age and 8.4% for those who did not have a partner. There was a relative decrease in the mean of 0.9% for each year of study.

Frailty had a relative increase in the mean of 3.3% for each additional disease, as well as 11.1% for the older adult who suffered a fall. In the final analysis, we verified a decrease of 0.011 points in the dispersion in the score of the frailty scale for each year of age and a decrease of 0.172 points in the dispersion for those who did not have a partner. There was a decrease of 0.0044 points in the dispersion of the frailty score for each additional point of the FIM. The opposite occurred in the evaluation using the Lawton and Brody Scale, with an increase of 0.0491 points in the dispersion for each IADL point (Table 3).

**Table 3:** Adjustment obtained by analysis of generalized estimation equation for the frailty score together with the parameters of dispersion. Ribeirão Preto, State of São Paulo, Brazil, 2016.

Variable	Estimate	Standard error	Wald	95%CI	p*	RI	95%CI
Intercept	0.948	0.164	33.170	0.626–1.271	< 0.001		
Age	0.005	0.002	8.576	0.001–0.009	0.003	1.005	1.001–1.009
Without partner	0.080	0.034	5.507	0.013–0.147	0.018	1.083	1.013–1.589
Education level	-0.008	0.003	5.470	-0.016– -0.001	0.019	0.991	0.983–0.998
Number of diseases	0.033	0.004	53.703	0.024–0.041	< 0.001	1.033	1.024–1.042
Number of drugs	0.009	0.005	3.175	-0.001–0.020	0.074	1.009	0.999–1.020
Suffered a fall	0.111	0.005	12.252	0.049–0.173	< 0.001	1.117	1.050–1.189
Linear total FIM	-2.302	0.434	28.123		< 0.001		
Quadratic total FIM	-0.547	0.297	3.383		0.065		
Linear total IADL	-4.466	0.509	76.871		< 0.001		
Quadratic total IADL	-2.053	0.295	48.371		< 0.001		
Period 2 (2013)	-0.114	0.032	12.592	-0.177– -0.051	< 0.001	0.892	0.837–0.950
Dispersion							
Intercept	1.352	0.424	10.174	0.521–2.183	0.001		
Age	-0.011	0.004	6.329	-0.020– -0.002	0.011		
Without partner	-0.172	0.083	4.236	-0.337– -0.008	0.039		
Total FIM	-0.004	0.002	4.375	-0.008– -0.000	0.036		
Total IADL	0.049	0.010	21.809	0.028–0.069	< 0.001		
Correlation	0.183	0.059	9.660		0.001		

FIM: functional independence measure; IADL: instrumental activities of daily living; RI: relative increase

* p ≤ 0.05

## DISCUSSION

Most participants (P2) were considered frail, with a predominance of females, older individuals (80 years or more), those without a partner, and those with low education level. We can observe differences in the means in the evaluation of older adults in both periods and the relation between frailty and age (older), marital status (no partner), and decreased functional capacity over time.

Mean frailty and the frailty category increased from P1 to P2. This finding is consistent with the study of Hyde et al.^19^ These researchers, when estimating the prevalence and incidence of frailty in older Australians, have observed that the prevalence of frailty increased from 65.3% to 67.6% and incidence reached 51.4% in 10 years^19^.

The frailty syndrome causes an accelerated decline in the physiological reserve, and homeostatic mechanisms begin to fail^2^ ^-^ ^21^ from the cumulative decline in the physiological system derived from mechanical complexes. There is consequent an erosion of the homeostatic reserve and vulnerability to disproportionate changes in health status after relatively minor stress events^22^. There is a continuous loss of strength and aerobic resistance, which causes a decrease in functional independence and makes the older adult frail^23^. This loss is related to increased age^24^, females, and low education level^25^.

In the dispersion analysis model, greater frailty was related to increased age. On the other hand, a study with 14,424 older adults in a two-year follow-up has observed an increase in frailty with increasing age, especially among those aged 75 years or more^26^.

The relation between age and frailty is due to the oxidative stress modulated by endogenous and exogenous agents influencing cellular oxygen production. This leads to DNA damage and causes changes in the cell with dysregulation in the inflammatory process, apoptosis, necrosis, and proliferation, which results in disadvantageous conditions, such as sarcopenia and fragility^8^ ^-^ ^27^.

Frailty was related to older adults who did not have a partner. Other studies have also presented this information^28^ ^,^ ^29^; the presence of a partner is a protective effect. The negative consequences on the physical and health status associated with the more limited family and social relationships of persons without partners increase the risk of social isolation^30^.

From P1 to P2, frailty was related to a decreased functional capacity in older adults. A study with 366 older adults evaluated with the Edmonton Frail Scale has shown that frailty was associated with Activities of Daily Living (ADL) (β = -0.512; p < 0.001) and IADL (β = -0.338; p < 0.01)^31^. On the other hand, a systematic review has pointed out that the older adult considered frail has a high risk of disability for ADL (OR = 2.76; 95%CI 2.33–3.44; p < 0.001), as well as for IADL (OR = 3.62; 95%CI 2.32–5.64; p < 0.001)^9^.

Frailty leads to decreased strength, weakness, and worsening motor performance. These characteristics are independent of the number of diseases that the older adult has^1^. Decreased functional capacity in frail older adults can result in high costs to health services. It is important to implement preventive actions against conditions related to different geriatric syndromes^8^. Scientific evidence suggests that the frailty syndrome can be changed as it is a dynamic process. Interventions such as physical exercise can reverse this phenomenon in older adults, depending on the duration of the intervention. This can decrease falls and improve mobility, balance, and muscle strength^32^.

Two limitations should be considered. The first one refers to losses from refusals, change of address to other cities, and death over time between P1 and P2, i.e., the study was restricted to survivors. The second one refers to how the study evaluated self-reported morbidities, and not medical diagnosis or medical records, which are used in epidemiological studies. Nevertheless, the data showed that the clinical evaluation points to an evolution of the frailty of older adults and its related factors. This indicates the need for the follow-up of older adults at home by the primary health care and the need for actions to prevent this syndrome and its adverse effects.

The primary care nurse should implement the use of low cost instruments for the preservation of functional independence, as well as the reduction of adverse events such as institutionalization and early mortality.

The evolution of the frailty syndrome in older adults was associated with increasing age, no partner, and decreased functional capacity in the follow-up years. However, this does not exclude other demographic and clinical variables from influencing the presence of this syndrome in older adults.

Instruments that can be easily used by nurses in primary care enable the identification of frailty in order to promote healthy and quality aging. Nevertheless, the government needs to invest in the creation of health policies focused on the promotion and education of young and mature adults to prevent frailty.
